# Are single-lumen 5Fr and triple-lumen 6Fr PICCs suitable for hemodynamic assessment by trans-pulmonary thermodilution? A pilot study

**DOI:** 10.1186/s13613-020-00785-2

**Published:** 2020-12-07

**Authors:** Sonia D’Arrigo, Claudio Sandroni, Sofia Cacciola, Antonio Maria Dell’Anna, Mauro Pittiruti, Maria Giuseppina Annetta, Cesare Colosimo, Massimo Antonelli

**Affiliations:** 1 Department of Intensive Care, Emergency Medicine and Anesthesiology, Fondazione Policlinico Universitario A. Gemelli-IRCCS, Largo Agostino Gemelli, 8, 00168 Rome, Italy; 2 Institute of Anesthesiology and Intensive Care Medicine, Università Cattolica del Sacro Cuore, Rome, Italy; 3 Department of Surgery, Fondazione Policlinico Universitario A. Gemelli-IRCCS, Università Cattolica del Sacro Cuore, Rome, Italy; 4 Department of Radiology, Fondazione Policlinico Universitario A. Gemelli-IRCCS, Università Cattolica del Sacro Cuore, Rome, Italy

**Keywords:** Peripherally inserted central catheter, Centrally inserted central catheter, Cardiac output, Trans-pulmonary thermodilution, Hemodynamic monitoring, Intensive care unit

## Abstract

**Background:**

Single-lumen 4Fr or double-lumen 5Fr power injectable peripherally inserted central catheters (PICCs) are not accurate for trans-pulmonary thermodilution (TPTD), since they overestimate cardiac index and other TPTD-derived parameters when compared with centrally inserted central catheters (CICCs) because of the smaller size of their lumen. We hypothesize that PICCs with larger lumen size may be reliable for the cardiac index assessment using the TPTD.

**Methods:**

This is a single-centre, prospective method–comparison study that included adult patients admitted in ICU who required a calibrated Pulse Contour hemodynamic monitoring system (VolumeView/EV1000™) for circulatory shock and had both PICC and CICC in place. We compared TPTD measurements via single-lumen 5Fr or triple-lumen 6Fr polyurethane power injectable PICCs with triple-lumen 7Fr CICC (reference standard). To rule out biases related to manual injection, measurements were repeated using an automated rapid injection system. We performed Bland–Altman analysis accounting for multiple observations per patient.

**Results:**

A total of 320 measurements were performed in 15 patients. During the manual phase, the cardiac index measured with either single-lumen 5Fr or triple-lumen 6Fr PICCs were comparable with cardiac index measured with triple-lumen 7Fr CICC (3.2 ± 1.04 vs. 3.2 ± 1.06 L/min/m^2^, bias 2.2% and 3.3 ± 0.8 vs. 3.0 ± 0.7 L/min/m^2^, bias 8.5%, respectively). During the automated phase, triple-lumen 6Fr PICC slightly overestimated the cardiac index when compared to triple-lumen 7Fr CICC (CI 3.4 ± 0.7 vs. 3.0 ± 0.7 L/min/m^2^, bias 12.5%; *p* = 0.012). For both single-lumen 5Fr and triple-lumen 6Fr PICCs, percentage error vs. triple-lumen 7Fr CICC was below 20% (14.7% and 19% during the manual phase and 14.4% and 13.8% during the automated phase, respectively). Similar results were observed for TPTD-derived parameters.

**Conclusions:**

During hemodynamic monitoring with TPTD, both single-lumen 5Fr PICCs and triple-lumen 6Fr PICCs can be used for cold fluid bolus injection as an alternative to CICC (ClinicalTrials.gov NCT04241926).

## Background

Power injectable polyurethane peripherally inserted central catheters (PICCs) allow high-speed fluid infusion (up to 3–5 mL/s), which makes them potentially suitable for cold fluid bolus injection during trans-pulmonary thermodilution (TPTD).

Several studies demonstrated that PICCs are equivalent to centrally inserted catheters (CICCs) for central venous pressure (CVP) measurement [1–6] but there is no evidence they can replace CICCs for cardiac output measurement using TPTD.

In a previous study from our group [7] we found that cold fluid bolus injection through a single-lumen 4Fr or a double-lumen 5Fr PICC for TPTD using VolumeView/EV1000™ significantly overestimated cardiac index (CI) and other TPTD-derived measures when compared with injection through the distal lumen of a 7Fr CICC. The most likely reason for this was the greater resistance to flow during bolus injection through these PICCs, due to their smaller size of their lumen, leading to an increased temperature of the fluid bolus and a consequent reduced difference (ΔT) between the temperature of the injected bolus and the blood temperature measured by the femoral arterial thermistor.

We hypothesized that using PICCs with a larger lumen size might reduce resistance to flow and ensure more accurate a measurement. The aim of our study was to evaluate whether triple-lumen 6Fr or single-lumen 5Fr PICCs could be as accurate as 7Fr CICCs (standard reference) for hemodynamic measurements with TPTD using the VolumeView/EV1000™ system.

## Methods

This study (ClinicalTrials.gov NCT04241926) was approved by the institutional review board (Prot. SF47489/18 ID1507). All patients or their legal representatives gave their written informed consent to participate.

The study was conducted in the general Intensive Care Unit (ICU) of the Fondazione Policlinico Universitario “A. Gemelli”-IRCCS Hospital, in Rome, Italy. Over a period of 6 months we considered for inclusion all adult patients (≥ 18 years) who required hemodynamic monitoring using TPTD because of hemodynamic instability (defined by a combination of vasopressor therapy needed to maintain a mean arterial pressure ≥ 65 mmHg and serum lactate above 2 mmol/L despite adequate fluid resuscitation) and had both a PICC and a CICC in place.

Some of these patients needed replacement of a PICC with a CICC at the time of ICU admission, or vice versa at the end of their ICU stay. Their enrolment occurred immediately after the placement of the new device and before the previous one was removed. Some patients requiring many separate lumens for drug infusion—typically during the early phases of septic shock—had simultaneously a CICC and a PICC in place for a few days.

Exclusion criteria were body weight < 40 kg (as from the manufacturer’s instructions for the TPTD system); severe right ventricular dysfunction; severe aortic regurgitation or intra-cardiac shunt; treatment with an intra-aortic balloon pump; contraindication to placement of PICC and/or CICC and/or femoral arterial catheter; abdominal aneurism; extracorporeal circulation; pregnancy; lack of informed consent. Further details about the protocol are included in our previous study [7].

We used single-lumen 5Fr or triple-lumen 6Fr power injectable polyurethane PICCs (Pro-PICC^®^ Medcomp, USA), inserted in deep veins of the upper arm, and triple-lumen 7Fr CICCs (Arrow G+ard Blue Plus^®^, Teleflex, USA), inserted in veins of the supra/infra-clavicular area.

Ultrasound-guided venipuncture using a 5–10 MHz linear ultrasound probe was adopted for both PICC and CICC insertion. For PICC placement, veins with ≥ 5 mm and ≥ 6 mm diameter were considered suitable for 5Fr and 6Fr catheters, respectively. When brachial or basilic veins of adequate size were not accessible in the middle third of the upper arm, 6Fr catheters were inserted in the axillary vein at the proximal third of the upper arm and then tunneled subcutaneously to have the exit site in the middle third.

The correct position of the tip of the venous catheters at the cavo-atrial junction was verified using the intracavitary electrocardiography (IC-ECG) method [8]. Tip location by post-procedural chest X-ray was used only when IC-ECG was not applicable. All catheters were secured using sutureless devices [9].

### Hemodynamic measurements

All triple-lumen 7Fr CICCs were 20 cm long and only the distal lumen was used for bolus injection. The length of single-lumen 5Fr and triple-lumen 6Fr PICCs depended on anthropometric patient’s characteristics. Only the largest lumen of triple-lumen 6Fr PICC was used for bolus injection.

1. *Manual phase (Phase 1):* in each patient, CI, global end-diastolic volume index (GEDVI), extravascular lung water index (ELWI), stroke volume index (SVI), and ΔT were assessed through manual injection of a 20-mL bolus of cold saline solution (4–6 °C) via both CICC and PICC. One single operator (SD’A) performed all measurements in all patients, to prevent any inter-observer variability.

2*. Automated rapid injector phase (Phase 2*): for each patient, to rule out potential biases related to manual injection, measurements were repeated using an automated rapid injection system (MEDRAD, Imaxeon, Bayer©). Bolus injection at a controlled speed of 5 mL/s was then performed and the pressure obtained by injection (pound per square inch-psi) was recorded.

During the measurements (about 15 min), the infusion rates of both sedatives and vasopressors and the ventilator settings were left unchanged. The head of the bed was maintained at 30° throughout the measurements.

### Statistical analysis

All data were included in a Microsoft Excel™ spreadsheet for record and analysis. Normal distributions were tested with Kolmogorov–Smirnov test. Continuous data were expressed as mean and standard deviation (SD). Differences between groups were assessed with parametric tests for independent samples or paired measures (i.e., one-way ANOVA or *t* test for paired measures), as appropriate.

Categorical data were reported as numbers and percentages (%) and their differences were assessed with Fisher’s exact test. Correlation of measures was explored with Spearman’s rho test. Bland–Altman analysis for multiple observations per individual was used to measure bias, the relevant lower and upper limits of agreement (LoA) [10], and the percentage error (PE) of the limits of agreement. This was calculated by dividing the LoA by the mean CI from the two methods, as reported by Critchley and Critchley [11].

Confidence intervals (CIs) were calculated as recommended by Zou et al. [12]. MedCalc Statistical Software version 19.2.1 (MedCalc Software Ltd, Ostend, Belgium). was used for calculation. Based on data from our previous study, we aimed at 80 couples of measurements, assuming for both 6-Fr and 5-Fr PICCs a reduced error in CI estimation of 0.4 with an alpha < 0.05 and a power of 95%.

## Results

### Demographic and clinical characteristics

**Fig. 1:**
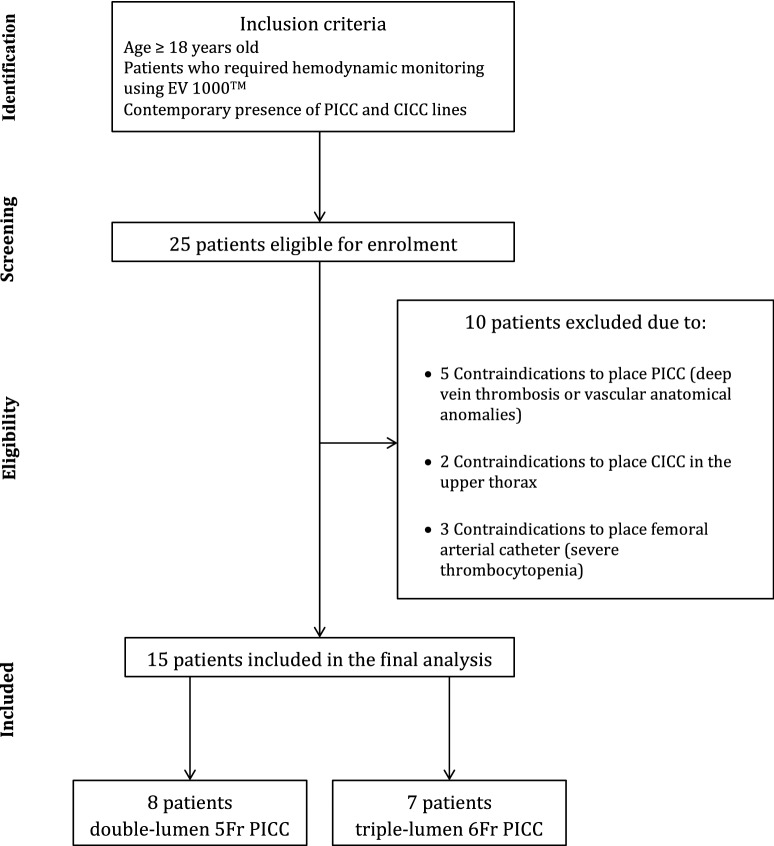
Flowchart of study population

Over a period of 6 months, 25 eligible patients were screened for inclusion. Of these, five were excluded because of contraindication to PICC placement (deep venous thrombosis or other vascular abnormalities), two were excluded because of contraindication to CICC placement, and three were excluded because of contraindications to femoral arterial catheter placement (severe thrombocytopenia), leaving 15 patients (8 males, 53.3%) for the final analysis (Fig. 1). Mean (SD) age was 62.5 (14.4) years, mean SAPS II was 59.8 (17.1) and mean body mass index was 26.3 (5.5) kg/m^2^.

PICCs were inserted via the brachial vein (three patients, 20%), the basilic vein (eight patients, 53.3%) or the axillary vein (four patients, 26.7%); in the latter group, the average length of the subcutaneous tunnel was 5.0 ± 1.1 cm.

Eight single-lumen 5Fr and seven triple-lumen 6Fr PICCs were placed. The average PICC length after insertion was 39.9 ± 3.0 cm (plus an extension length of 11.5 cm).

All triple-lumen 7Fr CICCs were 20 cm long (plus an extension length of 14 cm) and placed in the internal jugular vein (Table 1).

**Table 1:** Patient population: demographic and clinical characteristics

Characteristics	All patients (*n* = 15)
Age, years, mean (SD)	62.5 (14.4)
Male, *n* (%)	8 (53.3)
Body Mass Index, kg/m^2^, mean (SD)	26.3 (5.5)
SAPS II, mean (SD)	59.8 (17.1)
Diagnosis on admission in ICU, *n* (%)	
Septic shock	10 (66.6)
Acute respiratory failure	3 (20)
Cardiogenic shock	2 (13.4)
CICC characteristics	
Triple-lumen 7Fr, *n* (%)	15 (100)
Catheter length, cm	20
Extension length, cm	14
Approach, *n* (%)	
Internal jugular vein	15 (100)
PICC characteristics	
Single-lumen 5Fr, *n* (%)	8 (53.3)
Trimmed length 5Fr, cm, mean (SD)	40.6 (3)
Extension length 5Fr, cm	11.5
Triple-lumen 6Fr, *n* (%)	7 (46.6)
Trimmed length 6Fr, cm, mean (SD)	38.8 (2.9)
Extension length 6Fr, cm	11.5
Approach, *n* (%)	
Brachial vein	3 (20)
Basilic vein	8 (53.3)
Axillary vein	4 (26.7)

CICC: centrally inserted central catheter; ICU: intensive care unit; PICC: peripherally inserted central catheter; SAPS: simplified acute physiology score

Eighty couples of measurements were obtained after the injection via each of the two central venous devices (CICC and PICC) with both the manual phase and the automated rapid injector phase, for a total of 320 measurements.

### Manual phase

**Fig. 2:**
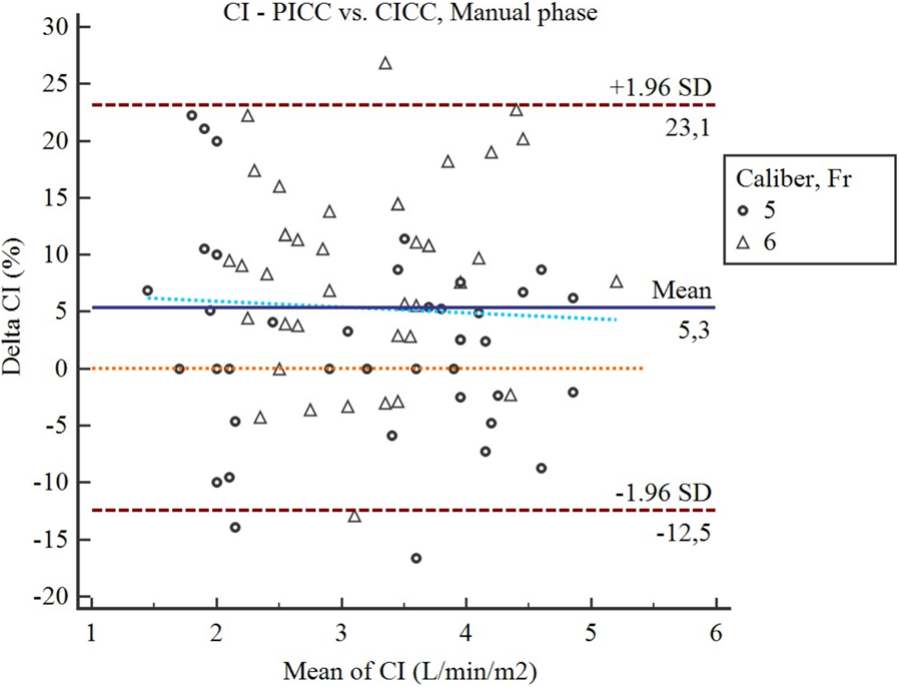
Bland-Altman plot comparing the difference of cardiac index between CICC and PICC during the manual phase. The solid horizontal line represents the mean bias (%) between the two devices. The two dashed horizontal lines correspond to the limits of agreement (mean bias ± 1.96 times the standard deviation of the differences)

During manual injection the mean CI when using single-lumen 5Fr PICC was identical to that obtained using triple-lumen 7Fr CICC (3.2 ± 1.04 vs. 3.2 ± 1.06 L/min/m^2^, bias 2.2%, PE 14.7%). Similarly, no differences in GEDVI, EVLWI, and SVI were found (Table 2; Fig. 2).

**Table 2:** Manual phase: Comparison between single-lumen 5Fr PICC vs. CICC

Variables	5 Fr single-lumen PICC	CICC	*p* value	Bias [LoA]	Bias, %	Error, %
Measurements, *n*	40	40	–	–	–	–
CI, L/min/m^2^	3.2 (1.04)	3.2 (1.06)	0.824	0.005 [− 0.42; 0.52]	2.2	14.7
GEDVI, mL/m^2^	716 (169)	694 (156)	0.539	22.5 [− 122; 167]	3.0	20.5
EVLWI, mL/kg	10.6 (2.5)	10.4 (2.9)	0.805	0.1 [− 2.0; 2.3]	2.5	21
SVI, mL/m^2^	36.1 (9.9)	35.0 (9.5)	0.582	1.2 [− 5.2; 7.6]	3.3	18
CVP, mmHg	10.1 (5.3)	10.1 (5.8)	0.823	–	–	–
ΔT, °C	0.32 (0.006)	0.32 (0.06)	0.611	− 0.007 [− 0.027; 0.041]	–	10.8

Data are reported as mean (standard deviation)

CI: cardiac index; CICC: centrally inserted central catheter; CVP: central venous pressure; EVLWI: extra-vascular lung water index; GEDVI: global end-diastolic volume index; LoA: limits of agreement; PICC: peripherally inserted central catheter; SVI: stroke volume index; Δ*T*: delta temperature

When using triple-lumen 6Fr PICC, the mean CI was comparable to that obtained using triple-lumen 7Fr CICC (3.3 ± 0.8 vs. 3.0 ± 0.7 L/min/m^2^, bias 8.5%, PE 19%) (Table 3; Fig. 2). Similar results were observed with the other hemodynamic variables.

**Table 3:** Manual phase: Comparison between triple-lumen 6Fr PICC vs. CICC

Variables	6 Fr triple-lumen PICC	CICC	*p* value	Bias [LoA]	Bias, %	Error, %
Measurements, *n*	40	40	–	–	–	–
CI, L/min/m^2^	3.3 (0.8)	3.0 (0.7)	0.107	0.28 [− 0.32; 0.88]	8.5	19
GEDVI, mL/m^2^	685 (133)	632 (102)	0.05	52.8 [− 75.7; 181]	7.4	19.5
EVLWI, mL/kg	14.0 (5.1)	12.2 (4.9)	0.178	1.5 [− 2.2; 5.2]	12.8	28.2
SVI, mL/m^2^	44.4 (10.4)	40.6 (8.5)	0.077	3.8 [− 4.2; 1.8]	8.5	18.8
CVP, mmHg	11.3 (4.8)	11.7 (5.5)	0.764	–	–	–
Δ*T*, °C	0.33 (0.07)	0.34 (0.08)	0.514	− 0.01 [− 0.06; 0.03]	–	12.9

Data are reported as mean (standard deviation)

CI: cardiac index; CICC: centrally inserted central catheter; CVP: central venous pressure; EVLWI: extra-vascular lung water index; GEDVI: global end-diastolic volume index; LoA: limits of agreement; PICC: peripherally inserted central catheter; SVI: stroke volume index; Δ*T*: delta temperature

#### ΔT and CVP

During manual injection, mean ΔT measured using single-lumen 5Fr PICCs was identical to that measured using CICCs (0.32 °C for both), whereas when using triple-lumen 6Fr PICCs ΔT was slightly lower than that measured using CICCs (0.33 vs. 0.34 °C) (Tables 2 and 3). No differences were found for measured CVP across the three catheters (Tables 2 and 3).

### Automated rapid injector phase

For single-lumen 5Fr PICC, the automated rapid injector phase confirmed the lack of any significant difference with triple-lumen 7Fr CICC for all hemodynamic variables (CI, GEDVI, EVLWI, SVI) and ΔT (Table 4).

**Table 4:** Automated injection phase: Comparison between single-lumen 5Fr PICC vs. CICC

Variables	5 Fr single-lumen PICC	CICC	*p* value	Bias [LoA]	Bias, %	Error, %
Measurements, *n*	40	40	–	–	–	–
CI, L/min/m^2^	3.2 (0.9)	3.06 (0.9)	0.44	0.16 [− 0.29; 0.61]	5.4	14.4
GEDVI, mL/m^2^	721 (171)	704 (188)	0.676	16.9 [− 83.2; 116.9]	3.1	14
EVLWI, mL/kg	11.4 (3.1)	10.9 (3.1)	0.487	0.49 [− 1.14; 2.12]	5.3	14.6
SVI, mL/m^2^	35.7 (8.5)	33.7 (8.6)	0.299	2.0 [− 3.2; 7.2]	6.1	15
CVP, mmHg	9.8 (5.6)	9.7 (4.5)	0.965	–	–	–
Δ*T*, °C	0.30 (0.06)	0.32 (0.06)	0.224	− 0.017 [− 0.045; 0.010]	–	8.7
Psi	112	109	0.07	–	–	–

Data are reported as mean (standard deviation)

CI: cardiac index; CICC: centrally inserted central catheter; CVP: central venous pressure; EVLWI: extra-vascular lung water index; GEDVI: global end-diastolic volume index; LoA: limits of agreement; PICC: peripherally inserted central catheter; Psi: pound per square inch; SVI: stroke volume index; Δ*T*: delta temperature

For triple-lumen 6Fr PICC, the measured CI was higher than that obtained using triple-lumen 7Fr CICC (3.4 ± 0.7 vs. 3.0 ± 0.7 L/min/m^2^, *p* = 0.012, bias 12.5%, PE 13.8%), and the same occurred to SVI and GEDVI. However, the PE was below 20% for all parameters (Table 5).

**Table 5:** Automated injection phase: Comparison between triple-lumen 6Fr PICC vs. CICC

Variables	6 Fr triple-lumen PICC	CICC	*p* value	Bias [LoA]	Bias, %	Error, %
Measurements, *n*	40	40	–	–	–	–
CI, L/min/m^2^	3.4 (0.7)	3.0 (0.7)	0.012	0.39 [− 0.05; 0.83]	12.5	13.8
GEDVI, mL/m^2^	689 (117)	622 (89)	0.006	66.5 [− 40; 173]	9.7	16.2
EVLWI, mL/kg	14.3 (5.0)	12.7 (4.5)	0.134	1.6 [− 0.4; 3.7]	12	15.6
SVI, mL/m^2^	44.6 (8.4)	39.4 (7.5)	0.004	5.2 [− 1.3; 11.7]	12.5	15.5
CVP, mmHg	12 (5.3)	11.1 (4.9)	0.458	–	–	–
Δ*T*, °C	0.30 (0.07)	0.34 (0.08)	0.008	− 0.05 [− 0.09; 0.00]	–	15.6
Psi	120	115	< 0.0001	–	–	–

Data are reported as mean (standard deviation)

CI: cardiac index; CICC: centrally inserted central catheter; CVP: central venous pressure; EVLWI: extra-vascular lung water index; GEDVI: global end-diastolic volume index; LoA: limits of agreement; PICC: peripherally inserted central catheter; Psi: pound per square inch; SVI: stroke volume index; Δ*T*: delta temperature

The pressure exerted during bolus injection through the single-lumen 5Fr PICC was similar to that measured through triple-lumen 7Fr CICC (112 vs. 109 psi; *p* = 0.07), whereas it was significantly higher when using triple-lumen 6 Fr PICC (120 vs. 115 psi; *p* < 0.0001) (Tables 4 and 5).

## Discussion

Our study showed that both single-lumen 5Fr PICCs and triple-lumen 6Fr PICCs are suitable for hemodynamic assessment using TPTD. Their PE was well below the 30% limit recommended for considering a new cardiac output measurement method as reliable [13]. However, unlike the 5Fr PICC, the triple-lumen 6Fr PICCs slightly overestimated CI when compared to triple-lumen 7Fr CICC. These results can be explained considering that the distal lumen of the 6Fr PICC is smaller than that of the 5Fr PICC (0.94 vs. 1.02 mm; see Table 6).

**Table 6:** Injection pressure, ΔT and CI during automated rapid injection through PICCs of different lumen size: comparison with CICC

	Triple-lumen 7Fr CICC	Single-lumen 5Fr PICC	Triple-lumen 6Fr PICC	Double-lumen 5Fr PICC^a^
Internal diameter, mm^a^	1.016	1.02	0.94	0.81
Mean Psi	110	112	120	130
ΔT bias, °C^b^	Reference	− 0.017	− 0.05	− 0.12
CI mean difference, L/min/m^2^	Reference	0.16	0.39	1.55
CI bias, %	Reference	5.4	12.5	39.3

^a^Data from D’Arrigo et al. [7]

^b^Difference between mean ΔT_PICC_ and mean ΔT_CICC_

CI: cardiac index; CICC: centrally inserted central catheter; ΔT: delta temperature; PICC: peripherally inserted central catheter; Psi: pound per square inch

In our previous study [7], we showed that CI and the other derived variables (GEDVI, SVI, EVLWI) were remarkably overestimated when double-lumen 5Fr or single-lumen 4Fr PICCs were used for bolus injection during TPTD, due to a significantly lower ΔT detected by the femoral arterial thermistor as compared to CICCs. The most likely cause is that a smaller catheter diameter is associated to a greater hydraulic resistance to flow during the injection, resulting in a greater dissipation of kinetic energy in the form of thermal energy and a consequent heating of the injected cold fluid bolus, which reduces the difference in temperature (ΔT) between the fluid bolus and the circulating blood. According to the Stewart–Hamilton equation, at a given volume and temperature of injected bolus, if the ΔT detected by the thermistor placed in femoral artery decreases, the measured CI increases [14]. An increase in the measured CI results in an overestimation of all the other TPTD variables, which depend on the thermodilution curve for their calculation.

Table 6, which includes data from both this and the previous study [7], shows that during the automated bolus injection, internal PICCs diameters were inversely related with injection pressure, ΔT bias and CI overestimation.

**Fig. 3:**
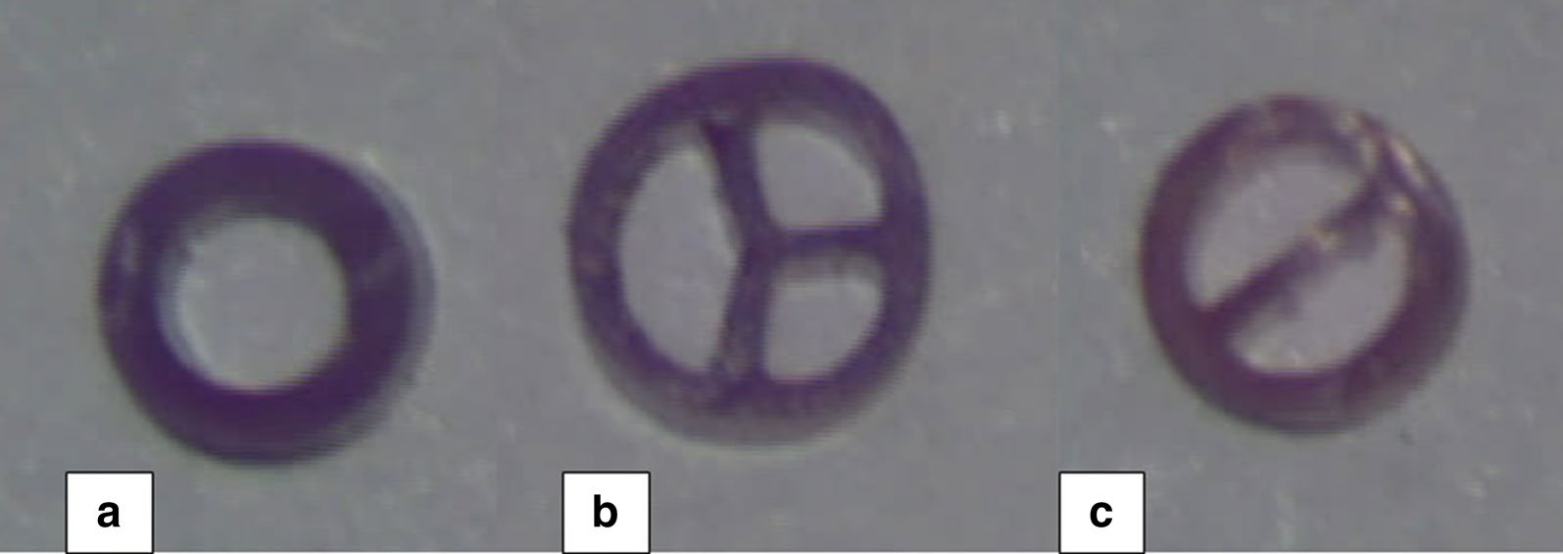
Cross section of catheters. A Single-lumen 5fr; B Triple-lumen 6Fr; C Double-lumen 5Fr

In addition to the smaller lumen size, the greater bias we observed for the triple-lumen 6Fr PICC vs. the single-lumen 5Fr PICC can also be attributed to its non-round shape (Fig. 3), which may contribute to the turbulence and the consequent heating of the thermodilution bolus. The slight CI overestimation associated with the use of the triple-lumen 6Fr PICC was more evident during the automated rapid injection phase, when the use of an automated injector ensured a more consistent injection time of the cold fluid bolus as compared to the manual phase.

The dependence of the bias we measured on the dynamic characteristics of the catheter used (i.e., resistance to bolus injection) is indirectly supported by the fact that in our study the CVP, which is a static pressure measured in no-flow conditions, was unaffected by the type of catheter used, as shown previously [1–6, 8].

Our study suggests that PICCs of adequate lumen size can replace CICCs for hemodynamic assessment using TPTD. This can be particularly useful when CICC placement is contraindicated or prone to complications, such as severe thrombocytopenia and/or coagulopathy [15, 16]. On the other side, triple-lumen PICC placement requires adequately large arm veins (≥ 6 mm). When these are not available, a proximal vein puncture and tunneling are needed, which require specific skills.

In our study, the bias associated with bolus injection through the single-lumen 5Fr PICC was negligible, which makes this catheter ideal for hemodynamic assessment. However, its single-lumen may limit its clinical applicability in critically ill patients when multiple simultaneous drug infusions are needed. In this case, the triple-lumen 6Fr PICC appears more suitable to replace the triple-lumen 7Fr CICC.

Our study has some limitations. First, we enrolled a relatively small number of patients since a pilot study. Though the total number of data was large enough to exclude a type II error according to our sample size calculation, a repeated error in the same patient may have affected our findings. Second, we used a 20-mL bolus for thermodilution. We do not know if using smaller boluses would have led to the same results. Third, CI was within the normal limits in most of our patients, which may potentially limit the external validity of our results in patients with more extreme values of CI.

## Conclusions

Our study showed that power injectable PICCs of adequate lumen size can be used as an alternative to CICCs for hemodynamic assessment using TPTD in adult ICU patients. Single-lumen 5Fr PICC were the most accurate when compared to CICC, while triple-lumen 6Fr PICC may be preferable when multiple infusion lumens are required.

## Data Availability

The dataset used and/or analyzed during the current study are available from the corresponding author on request. All data generated or analyzed during this study are included in this published article.

## Abbreviations

CI: Cardiac index
CICC: Centrally inserted central catheter
CIs: Confidence intervals
CVP: Central venous pressure
ELWI: Extravascular lung water index
GEDVI: Global end-diastolic volume index
IC-ECG: Intracavitary ECG
ICU: Intensive care unit
LoA: Limits of agreement
PICC: Peripherally inserted central catheter
Psi: Pound per square inch
SVI: Stroke volume index
TPTD: Trans-pulmonary thermodilution

## References

[1] Black IH, Blosser SA, Murray WB (2000). Central venous pressure measurements: peripherally inserted catheters versus centrally inserted catheters. Crit Care Med.

[2] Latham HE, Rawson ST, Dwyer TT, Patel CC, Wick JA, Simpson SQ (2012). Peripherally inserted central catheters are equivalent to centrally inserted catheters in intensive care unit patients for central venous pressure monitoring. J Clin Monit Comput.

[3] Latham HE, Dwyer TT, Gregg BL, Simpson SQ (2010). An in vitro study comparing a peripherally inserted central catheter to a conventional central venous catheter: no difference in static and dynamic pressure transmission. BMC Anesthesiol..

[4] McLemore EC, Tessier DJ, Rady MY, Larson JS, Mueller JT, Stone WM, Fowl RJ, Patel BM (2006). Intraoperative peripherally inserted central venous catheter central venous pressure monitoring in abdominal aortic aneurysm reconstruction. Ann Vasc Surg.

[5] Yun JY, Park SH, Cho DS, Jeung HJ, Lee SA, Seo SJ (2011). Comparison of the central venous pressure from internal jugular vein and the pressure measured from the peripherally inserted antecubital central catheter (PICCP) in liver transplantation recipients. Korean J Anesthesiol..

[6] Sanfilippo F, Noto A, Martucci G, Farbo M, Burgio G, Biasucci DG (2017). Central venous pressure monitoring via peripherally or centrally inserted central catheters: a systematic review and meta-analysis. J Vasc Access..

[7] D’Arrigo S, Sandroni C, Cacciola S, Dell’Anna AM, Pittiruti M, Annetta MG, Colosimo C, Antonelli M (2019). Are peripherally inserted central catheters suitable for cardiac output assessment with transpulmonary thermodilution?. Crit Care Med.

[8] Pittiruti M, La Greca A, Scoppettuolo G (2011). The electrocardiographic method for positioning the tip of central venous catheters. J Vasc Access..

[9] Yamamoto AJ, Solomon JA, Soulen MC, Tang J, Parkinson K, Lin R, Schears GJ (2002). Sutureless securement device reduces complications of peripherally inserted central venous catheters. J Vasc Interv Radiol..

[10] Bland JM, Altman DG (2007). Agreement between methods of measurement with multiple observations per individual. J Biopharm Stat.

[11] Critchley LA, Critchley JA (1999). A meta-analysis of studies using bias and precision statistics to compare cardiac output measurement techniques. J Clin Monit Comput.

[12] Zou GY (2013). Confidence interval estimation for the Bland-Altman limits of agreement with multiple observations per individual. Stat Methods Med Res.

[13] Odor PM, Bampoe S, Cecconi M (2017). Cardiac output monitoring: validation studies-how results should be psresented. Curr Anesthesiol Rep..

[14] Stewart GN (1897). Researches on the Circulation Time and on the Influences which affect it. The Journal of physiology..

[15] Pittiruti M, Brutti A, Celentano D, Pomponi M, Biasucci DG, Annetta MG, Scoppettuolo G (2012). Clinical experience with power-injectable PICCs in intensive care patients. Crit Care.

[16] Cotogni P, Pittiruti M (2014). Focus on peripherally inserted central catheters in critically ill patients. World J Crit Care Med..

